# Supplementary material for: A Novel Sphingosine Kinase Inhibitor Suppresses Chikungunya Virus Infection
Source: Viruses. 2022 May 24;14(6):1123. doi: 10.3390/v14061123 (PMC9229564; doi:10.3390/v14061123)

Supplementary Figure Legends

Supplementary Figure S1. A) Table showing SK inhibitors screened for anti-CHIKV activity along with isoform selectivity. B) Structure of screened compounds from C) along with references.

Supplementary Figure S2. SLL3071511 remains effective at higher CHIKV MOI. A) Representative images of SLL3071511 inhibition of CHIKV infection were collected using the Operetta CLS microscope (20x objective). HeLa cells were pre-treated for 1 hour at the indicated concentrations and then infected with CHIKV (MOI = 10) for 24 hours. Staining was done for viral nonstructural protein (nsP3, green), nucleus (Hoechst33342, blue), and cytoplasm (CellMask, red). Images were obtained on the Operetta CLS confocal microscope (20x objective), and analysis was done using the Harmony software. Data represent means (± SD) from one representative experiment of at least two independent experiments performed in duplicate. B) Percent viability of HeLa cells following CHIKV inhibition was calculated using mock-infected cells as reference.

Supplementary Figure S3. Viral glycoprotein expression is also decreased with SLL3071511 treatment. A) Representative images of SLL3071511 inhibition of CHIKV infection were collected using the Operetta CLS microscope (20x objective). HeLa cells were pre-treated for 1 hour at the indicated inhibitor concentrations and then infected with CHIKV (MOI = 1) for 24 hours. Staining was done for viral structural protein (E2, green), nucleus (Hoechst33342, blue), and cytoplasm (CellMask, red). Images were obtained on the Operetta CLS confocal microscope (20x objective), and analysis was done using the Harmony software. B) Percent viability of HeLa cells and inhibition of CHIKV infection was calculated using mock-infected cells as reference.

Supplementary Figure S1

A) Table identifying SK inhibitors screened for CHIKV inhibitory activity along with isoform selectivity

Identifier	Compound	Selectivity
1	SLC4011540	Dual
2	SLC4091423	SK2
3	SLC4091425	Unknown
4	SLC4101431	SK2
5	SLC5091612	Unknown
6	SLC5101464	Unknown
7	SLC5101465	SK1
8	SLC5111312	Dual
9	SLL3041783	SK1
10	SLL3071511	SK1
11	SLM6031434	SK2
12	SLM6071469	SK2
13	SLM6081456	Unknown
14	SLP101417	Unknown
15	SLP120701	SK2
16	SLP7111228	SK1
17	SLP9081411	Unknown
18	SLR080811	SK2
19	SLS1081832	Unknown

B) Structures of Sphingosine Kinase Inhibitors and associated publications.

Childress, E.; Kharel, Y.; Brown, A.; Bevan, D.R.; Lynch, K.R.; Santos, W.L. Transforming Sphingosine Kinase 1 Inhibitors into Dual and Sphingosine Kinase 2 Selective Inhibitors: Design, Synthesis, and In Vivo Activity. *J. Med. Chem.* **2017**, *60*, 3933-3957.

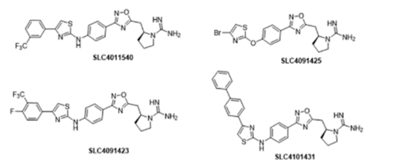

Congdon, M.; Fritzemeier, R.G.; Kharel, Y.; Brown, A.M.; Serbulea, V.; Bevan, D.R.; Lynch, K.R.; Santos, W.L. Probing the Substitution Pattern of Indole-Based Scaffold Reveal Potent and Selective Sphingosine Kinase 2 Inhibitors. *Eur. J. Med. Chem.* **2021,** *212*, 113121.

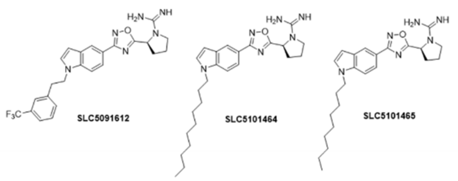

Congdon, M.; Kharel, Y.; Brown, A.; Lewis, S.L.; Bevan, D.R.; Lynch, K.R. and Santos, W.L. Structure-activity relationship studies and molecular modeling of naphthalene-based sphingosine kinase 2 inhibitors. *ACS Med. Chem. Lett.* **2016**, *7*, 229-234.

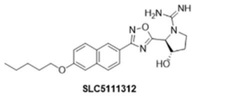

Li, H.; Sibley, C.D.; Kharel, Y.; Huang, T.; Brown, A.M.; Wonilowicz, L.G.; Bevan, D.R.; Lynch, K.R.; Santos, W.L. Lipophilic Tail Modifications of 2-(hydroxymethyl)pyrrolidine Scaffold Reveal Dual Sphingosine Kinase 1 and 2 Inhibitors. *Bioorg. Med. Chem.* **2021**, *30*, 115941.

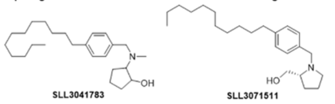

Sibley, C.D.; Morris, E.A.; Kharel, Y.; Brown, A.M.; Bevan, D.R.; Lynch, K.R.; Santos, W.L. Discovery of a Small Side Cavity in Sphingosine Kinase 2 that Enhances Inhibitor Potency and Selectivity, *J. Med. Chem.* **2020**, 63, 1178-1198.

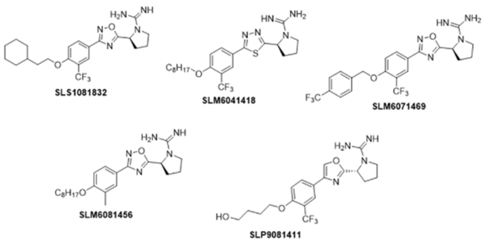

Supplementary Figure S2

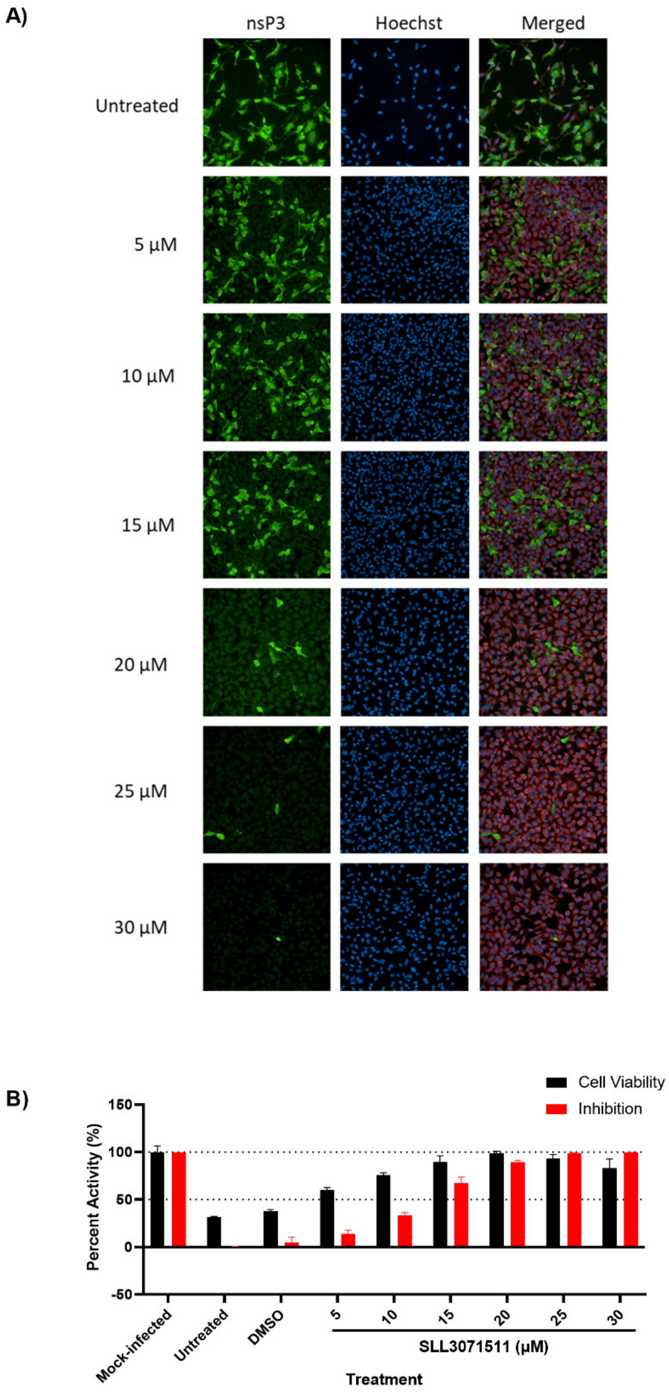

Supplementary Figure S3

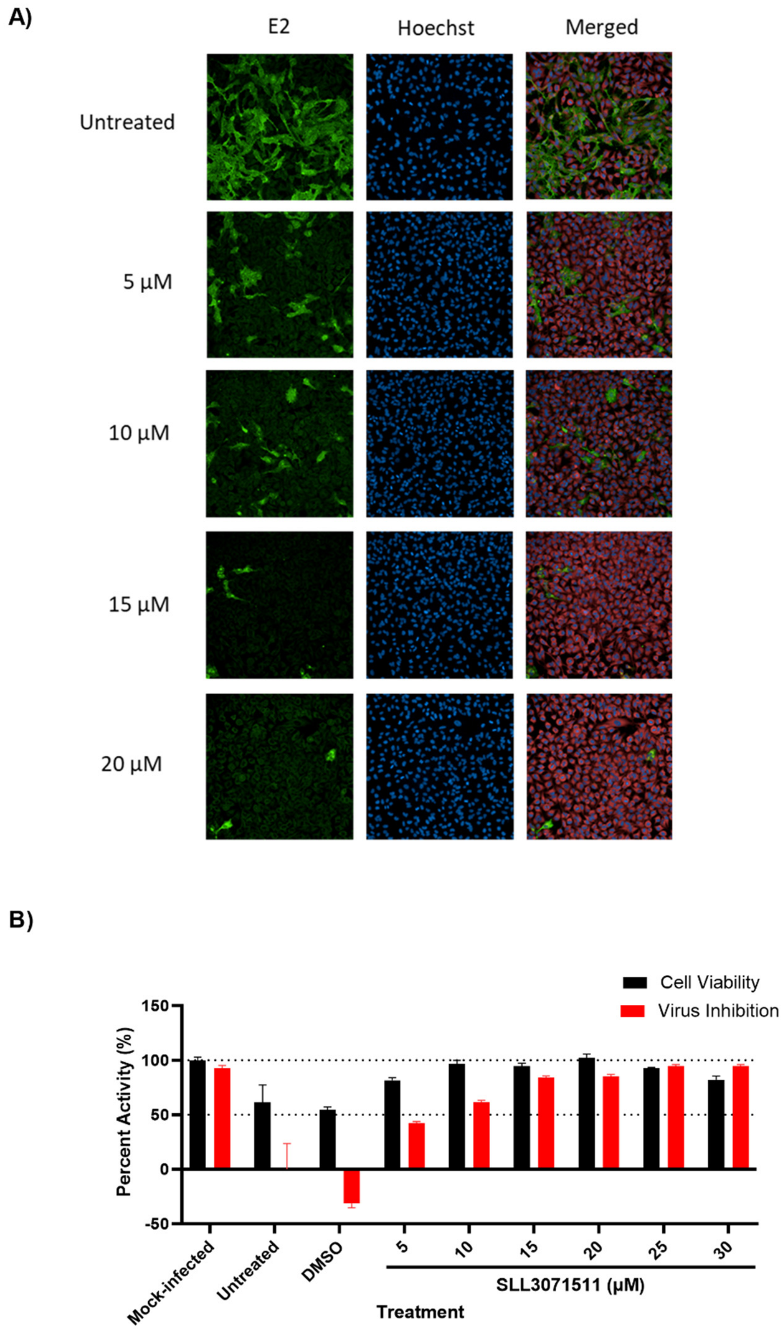

Supplement: Supplementary file 1 [file viruses-14-01123-s001.zip › viruses-1685426-supplementary.pdf]
